# Supplementary material for: Modeling glioblastoma heterogeneity as a dynamic network of cell states
Source: Mol Syst Biol. 2021 Sep 16;17(9):e10105. doi: 10.15252/msb.202010105 (PMC8444284; doi:10.15252/msb.202010105)
Supplement: Supplementary file 5 — Source Data for Figure 3 [file MSB-17-e10105-s001.zip › Figure3A_sourcedata/GSEA_3065/nosigdb_state2.GseaPreranked.1622718419215/DOROSZKO_U3013MG_CLUSTER_2.html]

Details for gene set DOROSZKO\_U3013MG\_CLUSTER\_2[GSEA]

|  || Dataset | state2 |
| Phenotype | NoPhenotypeAvailable |
| Upregulated in class | na\_pos |
| GeneSet | DOROSZKO\_U3013MG\_CLUSTER\_2 |
| Enrichment Score (ES) | 0.90966624 |
| Normalized Enrichment Score (NES) | 3.3030536 |
| Nominal p-value | 0.0 |
| FDR q-value | 0.0 |
| FWER p-Value | 0.0 |
Table: GSEA Results Summary

  

Fig 1: Enrichment plot: DOROSZKO\_U3013MG\_CLUSTER\_2      
 Profile of the Running ES Score & Positions of GeneSet Members on the Rank Ordered List

  

| PROBE | GENE SYMBOL | GENE\_TITLE | RANK IN GENE LIST | RANK METRIC SCORE | RUNNING ES | CORE ENRICHMENT || 1 | HIST1H4C |  |  | 0 | 1.309 | 0.0202 | Yes |
| 2 | PCLAF |  |  | 2 | 0.852 | 0.0333 | Yes |
| 3 | HMGB2 |  |  | 4 | 0.823 | 0.0459 | Yes |
| 4 | TYMS |  |  | 5 | 0.792 | 0.0581 | Yes |
| 5 | CENPU |  |  | 6 | 0.763 | 0.0699 | Yes |
| 6 | UBE2C |  |  | 9 | 0.704 | 0.0806 | Yes |
| 7 | H2AFX |  |  | 10 | 0.675 | 0.0910 | Yes |
| 8 | DHFR |  |  | 11 | 0.644 | 0.1009 | Yes |
| 9 | UBE2T |  |  | 12 | 0.643 | 0.1109 | Yes |
| 10 | MYBL2 |  |  | 14 | 0.600 | 0.1200 | Yes |
| 11 | TOP2A |  |  | 15 | 0.596 | 0.1292 | Yes |
| 12 | MKI67 |  |  | 17 | 0.590 | 0.1382 | Yes |
| 13 | CDK1 |  |  | 18 | 0.577 | 0.1471 | Yes |
| 14 | UBE2S |  |  | 19 | 0.574 | 0.1560 | Yes |
| 15 | MAD2L1 |  |  | 20 | 0.563 | 0.1647 | Yes |
| 16 | ATAD2 |  |  | 21 | 0.563 | 0.1734 | Yes |
| 17 | RRM2 |  |  | 22 | 0.556 | 0.1820 | Yes |
| 18 | CKS1B |  |  | 23 | 0.554 | 0.1905 | Yes |
| 19 | NUDT1 |  |  | 26 | 0.541 | 0.1987 | Yes |
| 20 | CLSPN |  |  | 27 | 0.536 | 0.2070 | Yes |
| 21 | CKS2 |  |  | 28 | 0.532 | 0.2152 | Yes |
| 22 | TPX2 |  |  | 29 | 0.531 | 0.2234 | Yes |
| 23 | ESCO2 |  |  | 30 | 0.530 | 0.2316 | Yes |
| 24 | FANCI |  |  | 32 | 0.521 | 0.2395 | Yes |
| 25 | CENPF |  |  | 34 | 0.518 | 0.2474 | Yes |
| 26 | RPA3 |  |  | 36 | 0.511 | 0.2552 | Yes |
| 27 | SMC2 |  |  | 39 | 0.493 | 0.2626 | Yes |
| 28 | ORC6 |  |  | 40 | 0.493 | 0.2702 | Yes |
| 29 | DBF4 |  |  | 41 | 0.492 | 0.2778 | Yes |
| 30 | SMC4 |  |  | 42 | 0.489 | 0.2853 | Yes |
| 31 | TMPO |  |  | 43 | 0.489 | 0.2929 | Yes |
| 32 | CENPK |  |  | 44 | 0.488 | 0.3004 | Yes |
| 33 | ZWINT |  |  | 45 | 0.488 | 0.3080 | Yes |
| 34 | PBK |  |  | 46 | 0.484 | 0.3154 | Yes |
| 35 | NUSAP1 |  |  | 47 | 0.483 | 0.3229 | Yes |
| 36 | ANP32B |  |  | 48 | 0.474 | 0.3302 | Yes |
| 37 | KPNA2 |  |  | 50 | 0.472 | 0.3374 | Yes |
| 38 | CENPM |  |  | 54 | 0.460 | 0.3442 | Yes |
| 39 | TK1 |  |  | 56 | 0.456 | 0.3511 | Yes |
| 40 | SGO1 |  |  | 58 | 0.449 | 0.3579 | Yes |
| 41 | NCAPG |  |  | 60 | 0.445 | 0.3647 | Yes |
| 42 | BIRC5 |  |  | 61 | 0.443 | 0.3715 | Yes |
| 43 | PTTG1 |  |  | 63 | 0.439 | 0.3782 | Yes |
| 44 | GMNN |  |  | 64 | 0.435 | 0.3849 | Yes |
| 45 | CCNB1 |  |  | 68 | 0.420 | 0.3911 | Yes |
| 46 | LMNB1 |  |  | 69 | 0.417 | 0.3975 | Yes |
| 47 | EZH2 |  |  | 70 | 0.417 | 0.4039 | Yes |
| 48 | CENPH |  |  | 71 | 0.412 | 0.4103 | Yes |
| 49 | RRM1 |  |  | 72 | 0.412 | 0.4167 | Yes |
| 50 | ASF1B |  |  | 76 | 0.407 | 0.4226 | Yes |
| 51 | SGO2 |  |  | 77 | 0.406 | 0.4289 | Yes |
| 52 | PIMREG |  |  | 80 | 0.398 | 0.4348 | Yes |
| 53 | KIF4A |  |  | 84 | 0.393 | 0.4406 | Yes |
| 54 | LSM5 |  |  | 85 | 0.393 | 0.4466 | Yes |
| 55 | KIF15 |  |  | 86 | 0.392 | 0.4527 | Yes |
| 56 | CENPN |  |  | 87 | 0.390 | 0.4587 | Yes |
| 57 | GTSE1 |  |  | 89 | 0.389 | 0.4646 | Yes |
| 58 | RAD51AP1 |  |  | 90 | 0.388 | 0.4706 | Yes |
| 59 | ALYREF |  |  | 91 | 0.382 | 0.4765 | Yes |
| 60 | BRCA1 |  |  | 92 | 0.381 | 0.4824 | Yes |
| 61 | PHF19 |  |  | 93 | 0.380 | 0.4883 | Yes |
| 62 | DTYMK |  |  | 94 | 0.379 | 0.4941 | Yes |
| 63 | MND1 |  |  | 96 | 0.376 | 0.4998 | Yes |
| 64 | CDKN3 |  |  | 97 | 0.376 | 0.5056 | Yes |
| 65 | TMSB15A |  |  | 99 | 0.374 | 0.5113 | Yes |
| 66 | CDCA5 |  |  | 101 | 0.373 | 0.5169 | Yes |
| 67 | DNAJC9 |  |  | 103 | 0.373 | 0.5226 | Yes |
| 68 | SPC25 |  |  | 104 | 0.372 | 0.5283 | Yes |
| 69 | CENPE |  |  | 105 | 0.372 | 0.5341 | Yes |
| 70 | FEN1 |  |  | 107 | 0.371 | 0.5397 | Yes |
| 71 | CACYBP |  |  | 109 | 0.366 | 0.5452 | Yes |
| 72 | RHEB |  |  | 110 | 0.362 | 0.5508 | Yes |
| 73 | ANLN |  |  | 111 | 0.358 | 0.5564 | Yes |
| 74 | NDC80 |  |  | 115 | 0.354 | 0.5615 | Yes |
| 75 | KIF23 |  |  | 117 | 0.351 | 0.5668 | Yes |
| 76 | POP7 |  |  | 119 | 0.347 | 0.5721 | Yes |
| 77 | ASPM |  |  | 122 | 0.342 | 0.5771 | Yes |
| 78 | CDKN2C |  |  | 123 | 0.341 | 0.5824 | Yes |
| 79 | TUBB4B |  |  | 125 | 0.340 | 0.5875 | Yes |
| 80 | CCDC34 |  |  | 127 | 0.339 | 0.5927 | Yes |
| 81 | PTGES3 |  |  | 128 | 0.336 | 0.5979 | Yes |
| 82 | KIF11 |  |  | 129 | 0.335 | 0.6030 | Yes |
| 83 | TACC3 |  |  | 130 | 0.334 | 0.6082 | Yes |
| 84 | MELK |  |  | 133 | 0.332 | 0.6131 | Yes |
| 85 | POC1A |  |  | 134 | 0.332 | 0.6182 | Yes |
| 86 | CCNA2 |  |  | 135 | 0.331 | 0.6233 | Yes |
| 87 | ANP32E |  |  | 136 | 0.330 | 0.6285 | Yes |
| 88 | USP1 |  |  | 138 | 0.329 | 0.6334 | Yes |
| 89 | ARHGAP11A |  |  | 145 | 0.321 | 0.6377 | Yes |
| 90 | RAD21 |  |  | 147 | 0.316 | 0.6425 | Yes |
| 91 | CDCA2 |  |  | 150 | 0.309 | 0.6471 | Yes |
| 92 | CKAP2 |  |  | 151 | 0.308 | 0.6518 | Yes |
| 93 | KIF22 |  |  | 154 | 0.306 | 0.6563 | Yes |
| 94 | CEP55 |  |  | 158 | 0.306 | 0.6607 | Yes |
| 95 | PSMC3IP |  |  | 159 | 0.305 | 0.6654 | Yes |
| 96 | CDCA3 |  |  | 160 | 0.304 | 0.6701 | Yes |
| 97 | AURKB |  |  | 163 | 0.303 | 0.6746 | Yes |
| 98 | SAP30 |  |  | 164 | 0.303 | 0.6793 | Yes |
| 99 | LMNB2 |  |  | 169 | 0.298 | 0.6834 | Yes |
| 100 | ECT2 |  |  | 171 | 0.297 | 0.6879 | Yes |
| 101 | BTG3 |  |  | 173 | 0.295 | 0.6924 | Yes |
| 102 | ITGB3BP |  |  | 177 | 0.294 | 0.6966 | Yes |
| 103 | DDX39A |  |  | 183 | 0.293 | 0.7006 | Yes |
| 104 | NUF2 |  |  | 188 | 0.287 | 0.7046 | Yes |
| 105 | CCNB2 |  |  | 190 | 0.286 | 0.7089 | Yes |
| 106 | CDC20 |  |  | 193 | 0.284 | 0.7131 | Yes |
| 107 | DEPDC1 |  |  | 196 | 0.283 | 0.7172 | Yes |
| 108 | HMGB3 |  |  | 200 | 0.282 | 0.7213 | Yes |
| 109 | FOXM1 |  |  | 202 | 0.282 | 0.7255 | Yes |
| 110 | KIF20B |  |  | 208 | 0.278 | 0.7293 | Yes |
| 111 | HJURP |  |  | 209 | 0.277 | 0.7335 | Yes |
| 112 | YWHAH |  |  | 221 | 0.271 | 0.7365 | Yes |
| 113 | ATAD5 |  |  | 222 | 0.269 | 0.7407 | Yes |
| 114 | CBX5 |  |  | 225 | 0.265 | 0.7446 | Yes |
| 115 | SHCBP1 |  |  | 231 | 0.262 | 0.7481 | Yes |
| 116 | JPT1 |  |  | 236 | 0.260 | 0.7516 | Yes |
| 117 | CENPA |  |  | 238 | 0.259 | 0.7555 | Yes |
| 118 | ILF2 |  |  | 240 | 0.258 | 0.7594 | Yes |
| 119 | PGP |  |  | 244 | 0.254 | 0.7630 | Yes |
| 120 | KIF2C |  |  | 252 | 0.251 | 0.7661 | Yes |
| 121 | CKLF |  |  | 254 | 0.249 | 0.7698 | Yes |
| 122 | SPC24 |  |  | 256 | 0.248 | 0.7736 | Yes |
| 123 | MIS18BP1 |  |  | 257 | 0.246 | 0.7774 | Yes |
| 124 | SAC3D1 |  |  | 263 | 0.246 | 0.7806 | Yes |
| 125 | PRR11 |  |  | 285 | 0.238 | 0.7820 | Yes |
| 126 | TRIP13 |  |  | 286 | 0.237 | 0.7857 | Yes |
| 127 | ACTL6A |  |  | 287 | 0.237 | 0.7893 | Yes |
| 128 | NCAPD3 |  |  | 294 | 0.234 | 0.7923 | Yes |
| 129 | KIF14 |  |  | 302 | 0.231 | 0.7951 | Yes |
| 130 | CIP2A |  |  | 304 | 0.230 | 0.7986 | Yes |
| 131 | BUB3 |  |  | 311 | 0.228 | 0.8014 | Yes |
| 132 | DIAPH3 |  |  | 321 | 0.226 | 0.8039 | Yes |
| 133 | CKAP2L |  |  | 327 | 0.224 | 0.8069 | Yes |
| 134 | MXD3 |  |  | 328 | 0.224 | 0.8103 | Yes |
| 135 | RACGAP1 |  |  | 333 | 0.222 | 0.8133 | Yes |
| 136 | BUB1 |  |  | 335 | 0.222 | 0.8166 | Yes |
| 137 | NCAPH |  |  | 336 | 0.222 | 0.8201 | Yes |
| 138 | HIRIP3 |  |  | 347 | 0.217 | 0.8223 | Yes |
| 139 | LSM8 |  |  | 354 | 0.215 | 0.8250 | Yes |
| 140 | MASTL |  |  | 357 | 0.215 | 0.8281 | Yes |
| 141 | HMMR |  |  | 358 | 0.215 | 0.8314 | Yes |
| 142 | PLK1 |  |  | 364 | 0.213 | 0.8342 | Yes |
| 143 | DLGAP5 |  |  | 371 | 0.211 | 0.8368 | Yes |
| 144 | CENPW |  |  | 373 | 0.211 | 0.8399 | Yes |
| 145 | ZWILCH |  |  | 374 | 0.210 | 0.8432 | Yes |
| 146 | AURKA |  |  | 375 | 0.210 | 0.8464 | Yes |
| 147 | KNL1 |  |  | 379 | 0.209 | 0.8493 | Yes |
| 148 | SKA2 |  |  | 421 | 0.195 | 0.8479 | Yes |
| 149 | CDK5RAP2 |  |  | 423 | 0.195 | 0.8508 | Yes |
| 150 | BCL7C |  |  | 429 | 0.193 | 0.8532 | Yes |
| 151 | NCAPD2 |  |  | 434 | 0.193 | 0.8558 | Yes |
| 152 | TROAP |  |  | 435 | 0.193 | 0.8588 | Yes |
| 153 | TEDC1 |  |  | 438 | 0.192 | 0.8615 | Yes |
| 154 | SPAG5 |  |  | 439 | 0.192 | 0.8645 | Yes |
| 155 | NSD2 |  |  | 444 | 0.191 | 0.8670 | Yes |
| 156 | FAM111A |  |  | 448 | 0.191 | 0.8696 | Yes |
| 157 | EXOSC8 |  |  | 456 | 0.189 | 0.8718 | Yes |
| 158 | SPDL1 |  |  | 459 | 0.189 | 0.8745 | Yes |
| 159 | RTKN2 |  |  | 463 | 0.188 | 0.8771 | Yes |
| 160 | CENPQ |  |  | 472 | 0.185 | 0.8790 | Yes |
| 161 | KNSTRN |  |  | 483 | 0.183 | 0.8808 | Yes |
| 162 | CDKN2D |  |  | 499 | 0.182 | 0.8820 | Yes |
| 163 | HNRNPH3 |  |  | 504 | 0.180 | 0.8843 | Yes |
| 164 | PRC1 |  |  | 514 | 0.178 | 0.8861 | Yes |
| 165 | BCL2L12 |  |  | 517 | 0.178 | 0.8886 | Yes |
| 166 | CDC25B |  |  | 519 | 0.178 | 0.8913 | Yes |
| 167 | MAGOH |  |  | 542 | 0.174 | 0.8916 | Yes |
| 168 | KHDRBS1 |  |  | 548 | 0.173 | 0.8937 | Yes |
| 169 | KRBOX1 |  |  | 562 | 0.170 | 0.8949 | Yes |
| 170 | HDGF |  |  | 570 | 0.168 | 0.8967 | Yes |
| 171 | BUB1B |  |  | 572 | 0.168 | 0.8992 | Yes |
| 172 | KIFC1 |  |  | 577 | 0.167 | 0.9014 | Yes |
| 173 | PARPBP |  |  | 594 | 0.164 | 0.9022 | Yes |
| 174 | CKAP5 |  |  | 596 | 0.164 | 0.9046 | Yes |
| 175 | CBR3 |  |  | 616 | 0.161 | 0.9050 | Yes |
| 176 | RFC3 |  |  | 617 | 0.161 | 0.9075 | Yes |
| 177 | CDCA8 |  |  | 634 | 0.158 | 0.9082 | Yes |
| 178 | TUBA1C |  |  | 644 | 0.157 | 0.9097 | Yes |
| 179 | MT2A |  |  | 731 | 0.146 | 0.9026 | No |
| 180 | FAM83D |  |  | 759 | 0.143 | 0.9019 | No |
| 181 | MNS1 |  |  | 774 | 0.142 | 0.9026 | No |
| 182 | SKA3 |  |  | 790 | 0.139 | 0.9031 | No |
| 183 | NEK2 |  |  | 805 | 0.138 | 0.9037 | No |
| 184 | TTK |  |  | 831 | 0.135 | 0.9031 | No |
| 185 | PSRC1 |  |  | 1023 | 0.118 | 0.8842 | No |
| 186 | PPP2CA |  |  | 1044 | 0.116 | 0.8838 | No |
| 187 | KIF20A |  |  | 1094 | 0.112 | 0.8802 | No |
| 188 | HP1BP3 |  |  | 1249 | 0.102 | 0.8651 | No |
| 189 | CDC27 |  |  | 1319 | 0.097 | 0.8591 | No |
| 190 | PHIP |  |  | 1505 | 0.086 | 0.8404 | No |
| 191 | CCP110 |  |  | 1554 | 0.084 | 0.8365 | No |
| 192 | WEE1 |  |  | 1617 | 0.081 | 0.8311 | No |
| 193 | REEP4 |  |  | 1734 | 0.076 | 0.8197 | No |
| 194 | XPO1 |  |  | 1871 | 0.070 | 0.8060 | No |
| 195 | NEURL1B |  |  | 1929 | 0.067 | 0.8009 | No |
| 196 | GGH |  |  | 2149 | 0.059 | 0.7781 | No |
| 197 | OIP5 |  |  | 2175 | 0.058 | 0.7762 | No |
| 198 | G2E3 |  |  | 2405 | 0.051 | 0.7522 | No |
| 199 | SMTN |  |  | 2590 | 0.045 | 0.7330 | No |
| 200 | AC026401.3 |  |  | 2688 | 0.042 | 0.7231 | No |
| 201 | PSMD10 |  |  | 3415 | 0.026 | 0.6449 | No |
| 202 | POLR2A |  |  | 3680 | 0.021 | 0.6166 | No |
| 203 | C21orf58 |  |  | 3849 | 0.018 | 0.5987 | No |
| 204 | SOGA1 |  |  | 3851 | 0.018 | 0.5988 | No |
| 205 | UACA |  |  | 3925 | 0.017 | 0.5912 | No |
| 206 | GPSM2 |  |  | 5127 | -0.002 | 0.4611 | No |
| 207 | LBR |  |  | 7064 | -0.038 | 0.2520 | No |
| 208 | CAPS |  |  | 7927 | -0.064 | 0.1596 | No |
| 209 | NUCB2 |  |  | 8258 | -0.080 | 0.1251 | No |
| 210 | SYPL1 |  |  | 9182 | -0.185 | 0.0279 | No |
Table: GSEA details [plain text format]

  

Fig 2: DOROSZKO\_U3013MG\_CLUSTER\_2: Random ES distribution      
 Gene set null distribution of ES for **DOROSZKO\_U3013MG\_CLUSTER\_2**
